# IL-10-Engineered Dendritic Cells Modulate Allogeneic CD8^+^ T Cell Responses

**DOI:** 10.3390/ijms24119128

**Published:** 2023-05-23

**Authors:** Marta Fortunato, Giada Amodio, Silvia Gregori

**Affiliations:** 1 Mechanisms of Peripheral Tolerance Unit, San Raffaele Telethon Institute for Gene Therapy (SR-TIGET), IRCCS San Raffaele Scientific Institute, 20132 Milan, Italy; fortunato.marta@hsr.it (M.F.); amodio.giada@hsr.it (G.A.); 2 PhD Course in Molecular Medicine, University Vita-Salute San Raffaele, 20132 Milan, Italy

**Keywords:** dendritic cells, IL-10, CD8^+^ T cells, allogeneic responses

## Abstract

Tolerogenic dendritic cells (tolDC) play a central role in regulating immune homeostasis and in promoting peripheral tolerance. These features render tolDC a promising tool for cell-based approaches aimed at inducing tolerance in T-cell mediated diseases and in allogeneic transplantation. We developed a protocol to generate genetically engineered human tolDC overexpressing IL-10 (DC^IL-10^) by means of a bidirectional lentiviral vector (LV) encoding for IL-10. DC^IL-10^ promote allo-specific T regulatory type 1 (Tr1) cells, modulate allogeneic CD4^+^ T cell responses in vitro and in vivo, and are stable in a pro-inflammatory milieu. In the present study, we investigated the ability of DC^IL-10^ to modulate cytotoxic CD8^+^ T cell responses. We demonstrate that DC^IL-10^ reduces allogeneic CD8^+^ T cell proliferation and activation in primary mixed lymphocyte reactions (MLR). Moreover, long-term stimulation with DC^IL-10^ induces allo-specific anergic CD8^+^ T cells without signs of exhaustion. DC^IL-10^-primed CD8^+^ T cells display limited cytotoxic activity. These findings indicate that stable over-expression of IL-10 in human DC leads to a population of cells able to modulate cytotoxic allogeneic CD8^+^ T cell responses, overall indicating that DC^IL-10^ represent a promising cellular product for clinical applications aimed at inducing tolerance after transplantation.

## 1. Introduction

Dendritic cells (DC) are antigen-presenting cells (APC) able to induce naïve T cell activation and differentiation. In homeostatic conditions, DC are also involved in the induction and maintenance of immune tolerance. Indeed, DC at an immature stage or specialized subsets of DC, named tolerogenic DC (tolDC), control T-cell responses via several immunosuppressive mechanisms (reviewed in [1]) and induce the expansion and/or induction of regulatory T cells [2,3,4,5]. Exploiting the immune-regulatory capacities of DC holds great promise for the treatment of autoimmune diseases [6] and the prevention of graft rejection [7]. Several protocols have been established to efficiently differentiate tolDC in vitro starting from human peripheral blood monocytes (reviewed in [8]). A comparative analysis of different populations of in vitro differentiated tolDC suggested that IL-10-modulated DC are the most adequate cells for tolerance-inducing therapies [9]. Two prominent protocols to generate tolDC in the presence of IL-10 have been established. The first involves the exposure of immature DC to IL-10 in the last two days of DC differentiation [10], while the second entails the addition of IL-10 from the beginning of DC differentiation (DC-10) [11]. The latter cells are characterized by a unique cytokine production profile, the ability to spontaneously secrete IL-10 in the absence of IL-12, and by the co-expression of the tolerogenic molecules HLA-G and ILT4 [12]. These are critical features that render DC-10 potent inducers of T-cell anergy and allogeneic (allo)-specific T regulatory type 1 (Tr1) cells [11]. More recently, we developed an efficient protocol to generate IL-10-producing DC by bidirectional lentiviral vector (LV)-mediated IL-10 transduction of monocytes during DC differentiation (DC^IL-10^) [13]. DC^IL-10^ recapitulate the tolerogenic features of DC-10 as they secrete supraphysiological levels of IL-10, are stable upon exposure to pro-inflammatory signals, modulate allogeneic CD4^+^ T cells, and induce allo-specific Tr1 cell differentiation in vitro. We previously reported that DC^IL-10^ inhibit the proliferation of CD8^+^ T cells in primary cell culture [13], but detailed characterizations of DC^IL-10^-conditioned CD8^+^ T cells is still elusive.

CD8^+^ T cells, also known as cytotoxic T lymphocytes (CTL), are part of the adaptive immune system and play a crucial role in the clearance of intracellular pathogens and tumors. During the primary response, naive CD8^+^ T cells recognize antigen(Ag)s in the context of MHC class I molecules and undergo a differentiation process leading to strong clonal expansion to generate large numbers of cytotoxic effector CD8^+^ T cells. These cells migrate to the site of infection and mount an Ag-specific response (reviewed in [14,15,16]) to directly kill target cells through the release of cytotoxic molecules (e.g., perforin and granzymes) or via Fas–FasL interaction (reviewed in [17]). Moreover, cytotoxic effector CD8^+^ T cells secrete interferon (IFN)γ and tumor necrosis factor (TNF)-α [18,19], two important mediators for coordinating the innate and adaptive immune response. The CD8^+^ T cell activation process requires three signals provided by APC, primarily by DC: the engagement of TCR by peptide–MHC-I complexes, which defines the Ag specificity [20]; co-stimulatory signals provided by CD80/CD86 molecules [21,22]; and polarizing signal mediated by DC-derived cytokines, mainly IL-12 [23,24]. Additional stimulatory signals can be provided by CD4^+^ T helper cells, reinforcing DC-mediated activation and inducing the establishment of long-lasting memory CD8^+^ T cells [25,26].

In the present study, we investigated the ability of DC^IL-10^ to modulate allogeneic CD8^+^ T cell responses using short-term and long-term mixed lymphocyte reactions (MLR). Our findings revealed that DC^IL-10^ regulate allogeneic CD8^+^ T cell activation and proliferation in short-term culture, while in long-term co-culture, they induce anergic allo-specific CD8^+^ T cells, which display limited allo-specific cytotoxicity. Overall, DC^IL-10^ represent an interesting and effective tool for modulating not only CD4^+^, but also CD8^+^ allo-reactive T cell responses.

## 2. Results

### 2.1. DC^IL-10^ Modulate Allogeneic CD8^+^ T Cell Priming

**Figure 1:**
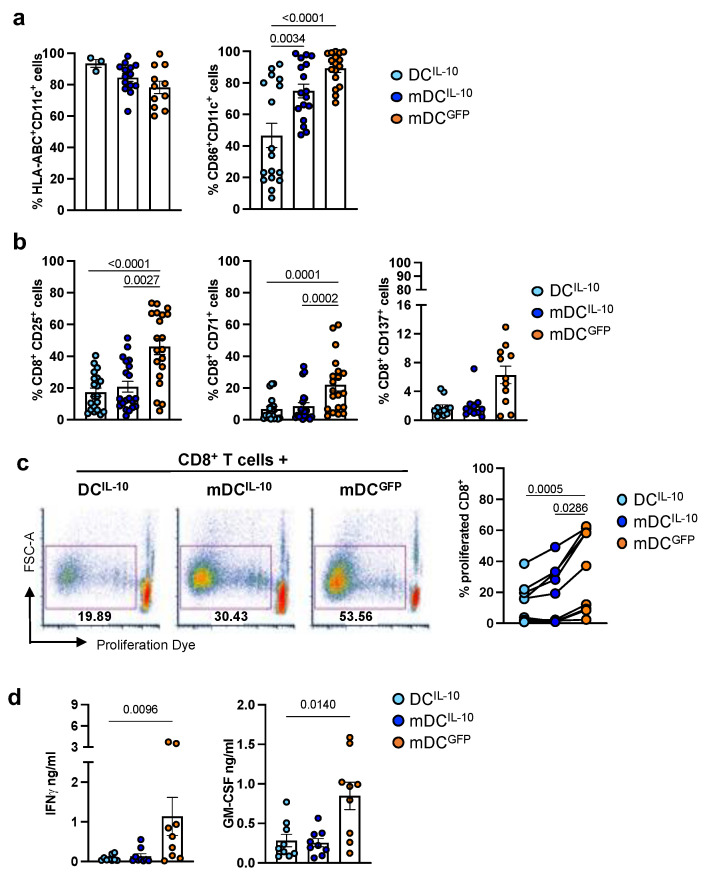
DC^IL-10^ modulate allogeneic CD8^+^ T cell responses. CD14^+^ cells isolated from peripheral blood of healthy subjects were transduced during DC differentiation with LV-IL-10 and left unstimulated (DC^IL-10^) or activated with LPS (mDC^IL-10^). As a control, DC transduced with LV-GFP and activated with LPS (mDC^GFP^) were differentiated from the same donors. (**a**) At the end of the differentiation, the expression of HLA-class I molecules (HLA-ABC, n = 3–14) and CD86 (n = 17) was evaluated by flow cytometry. Each dot represents a single donor; bars indicate mean ± SEM. (**b**–**d**) DC^IL-10^ inhibited the activation and proliferation of allogenic CD8^+^ T cells in short-term primary MLR. Allogeneic CD8^+^ T cells isolated from the peripheral blood of healthy subjects were stained with proliferation dye and stimulated with the indicated DC at a 10:1 ratio for 5 days. (**b**) After culture, CD8^+^ T cells were collected, and the expression of the activation markers CD25 (n = 20), CD71 (n = 20), and CD137 (n = 11) was evaluated by flow cytometry. Each dot represents a single donor; bars indicate mean ± SEM. (**c**) On day 5, the percentage of proliferated CD8^+^ cells was evaluated by proliferation dye dilution. Dot plots from one representative donor are presented (left panel); numbers indicate the percentage of proliferated cells. Percentage of proliferated CD8^+^ T cells stimulated with DC^IL-10^ (light blue dots), mDC^IL-10^ (blue dots), and mDC^GFP^ (orange dots) are shown (right panel). Each dot represents a single donor (n = 9). (**d**) IFNγ and GM-CSF were evaluated by ELISA in cell culture supernatants (n = 9). Each dot represents a single donor; bars indicate mean ± SEM. For all the statistical analyses, the Friedman matched-paired test, in association with Dunn’s multiple comparison test, was applied. Statistically significant *p* values are reported.

We investigated the ability of IL-10-engineered DC to modulate allogeneic CD8^+^ T cell responses in vitro. We generated immature IL-10-transduced DC (DC^IL-10^) or LPS-activated DC^IL-10^ (mDC^IL-10^) and compared them with LPS-activated GFP-transduced DC (mDC^GFP^). DC^IL-10^ and mDC^IL-10^ were efficiently transduced, based on the expression of the marker gene ΔNGFR (91.5 ± 1.1% and 93.5 ± 1.3%, of CD11c^+^ΔNGFR^+^ cells, n= 22 respectively), and expressed CD14, CD16, CD141, CD163, HLA-G, and ILT4 (Supplementary Figure S1a). Since efficient stimulation of CD8^+^ T cells requires the expression of MHC class I and costimulatory molecules [27], we evaluated their expression on DC^IL-10^, mDC^IL-10^, and mDC^GFP^, which showed that HLA-Class I was expressed at high and comparable levels (Figure 1a). CD86 was expressed by DC^IL-10^ and mDC^IL-10^ (46.6 ± 7.7% and 74.9 ± 4.4%, respectively, n = 22; Figure 1a), but, as previously observed [13], at variable levels among the donors tested. Despite this variability, DC^IL-10^ and mDC^IL-10^ expressed CD86 at significantly lower (*p* = 0.0034 and *p* < 0.0001, respectively) levels compared with mDC^GFP^ (89.2 ± 2.5%, n = 22; Figure 1a). 

As previously reported, activation of DC^IL-10^ with LPS did not alter their ability to secrete high levels of IL-10 [13]. Indeed, DC^IL-10^ and mDC^IL-10^ constitutively secreted above 30 ng/mL of IL-10, with mDC^IL-10^ being significantly (*p* = 0.0040) less efficient at producing IL-10 than DC^IL-10^ (159.7 ± 33.1 and 118.2 ± 26.4 ng/mL, respectively, n = 22; Supplementary Figure S1b). Despite these differences, allogeneic CD8^+^ T cells stimulated with DC^IL-10^ or mDC^IL-10^ expressed significantly (*p* < 0.0001 and *p* = 0.002, respectively) lower levels of the activation markers CD25 (17.4 ± 2.6% and 20.8 ± 3.4%, respectively, vs. 46.1 ± 5.0%, n = 20), CD71 (6.8 ± 1.6% and 8.5 ± 2.3% vs. 22 ± 3.9%, n = 20), and CD137 (17.0 ± 2.6% and 20.8 ± 3.4% vs. 46.1 ± 5%, n = 11) compared with T cells activated with mDC^GFP^ (Figure 1b). Accordingly, DC^IL-10^ and mDC^IL-10^ induced significantly (*p* = 0.0005 and *p* = 0.0286, respectively) lower proliferative responses in allogeneic CD8^+^ T cells compared with that elicited by mDC^GFP^ (Figure 1c). In line with this, CD8^+^ T cells co-cultured with DC^IL-10^ or mDC^IL-10^ secreted lower levels of IFNγ (0.080 ± 0.025 and 0.136 ± 0.063 ng/mL ng/mL, respectively, n = 9) compared with mDC^GFP^ (1.135 ± 0.480 ng/mL, n = 9), with the change in DC^IL-10^ reaching statistical significance (*p* = 0.0096) (Figure 1d). T cells co-cultured with DC^IL-10^ or mDC^IL-10^ also secreted lower levels of GM-CSF (0.283 ± 0.079 and 0.256 ± 0.053 ng/mL, respectively, n = 9), compared with those cultured with mDC^GFP^ (0.848 ± 0.173 ng/mL, n = 9) (Figure 1d). Although the secretion of GM-CSF from CD8^+^ T cells cultured with mDC^GFP^ was highly variable, the decrease in GM-CSF observed in CD8^+^ T cells cultured with DC^IL-10^ was statistically different (*p* = 0.0140) (Figure 1d). 

Overall, these findings indicate the both DC^IL-10^ at steady state and upon LSP activation modulate allogeneic CD8^+^ T cell responses.

To further characterize the modulatory activity of IL-10-engineered DC, we limited the comparison between mDC^IL-10^ and mDC^GFP^ to cells expressing similarly high levels of CD86 (Figure 1a). We first assessed the ability of CD8^+^ T cells primed with mDC^IL-10^ (T(mDC^IL-10^) cells) to release cytotoxic granules upon restimulation with mature DC (mDC) generated from the same donor used for priming (Supplementary Figure S2a). We observed an overall lower frequency of cells expressing granzyme (Gz)B and perforin (Prf) in T(mDC^IL-10^) cells (23.3 ± 1.7%, and 6.3 ± 2.5%, respectively, n = 5) compared with T cells stimulated with mDC^GFP^ (T(mDC^GFP^) cells) (54.4 ± 6.9% and 24.8 ± 12.6%, respectively, n = 5; Supplementary Figure S2b). Despite differences in the overall granule content, upon restimulation with mDC, the percentage of CD8^+^ T cells actively degranulating, as indicated by the percentage of GzB^+^CD107a^+^ or Prf^+^CD107a^+^ cells [28], was similar to that observed in unstimulated T cells for both T(mDC^IL-10^) and T(mDC^GFP^) cells (Supplementary Figure S2c,d). These findings suggest that short-term stimulation with allogeneic mDC^IL-10^ or mDC^GFP^ is not sufficient to induce fully competent cytotoxic CD8^+^ T cells.

### 2.2. DC^IL-10^ Induce Allo-Specific Anergic CD8^+^ T Cells

**Figure 2:**
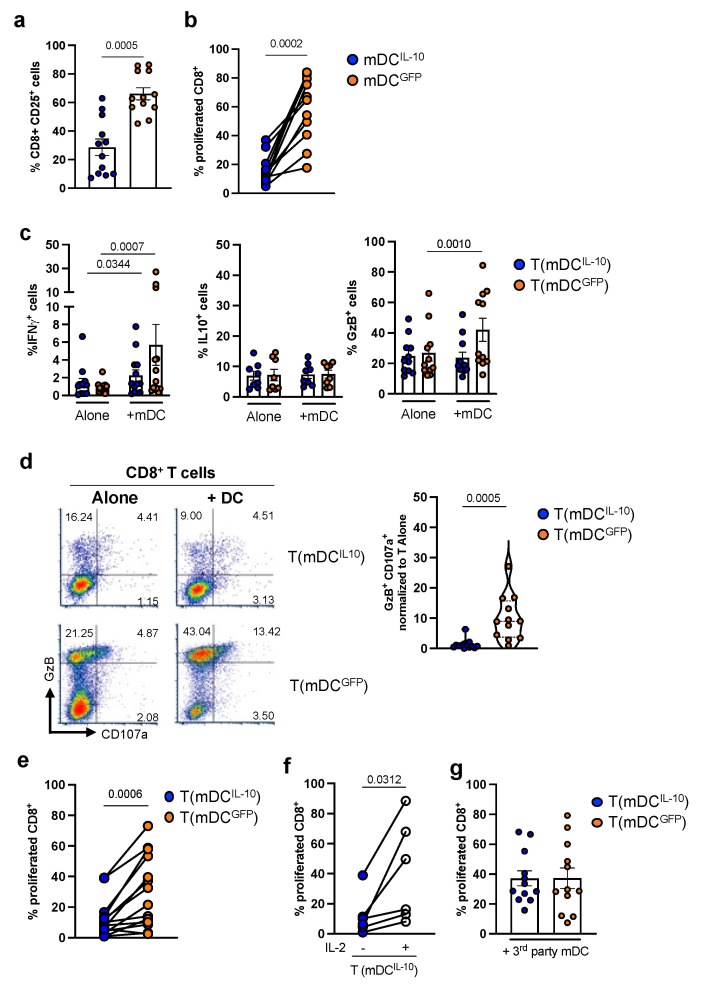
mDC^IL-10^ induce allo-specific anergic CD8^+^ T cells. CD14^+^ cells isolated from the peripheral blood of healthy subjects were transduced with LV-IL-10 or with LV-GFP and activated with LPS (mDC^IL-10^ and mDC^GFP^, respectively). Differentiated DC were used to stimulate at a 10:1 (T:DC) ratio of allogeneic CD8^+^ T cells labelled with proliferation dye for 14 days (primary MLR long-term). (**a**,**b**) mDC^IL-10^ modulated allogeneic CD8^+^ T cell responses in long-term primary MLR. After 14 days of culture, CD8^+^ T cells generated with mDC^IL-10^ or mDC^GFP^ were collected, and (**a**) the expression of the activation marker CD25 (n = 12) and (**b**) the percentage of proliferated CD8^+^ cells (n = 13) were evaluated by flow cytometry. Each dot represents a single donor (n = 12); bars indicate mean ± SEM. A two-tailed Wilcoxon matched-paired test was applied, and statistically significant *p* values are reported. (**c**,**d**) CD8^+^ T cells generated with mDC^IL-10^ T(mDC^IL-10^) or mDC^GFP^ T(mDC^GFP^) were collected and left un-stimulated or restimulated with mDC generated from the same donor used in priming, and the frequency of IFNγ^+^, IL-10^+^ and GzB^+^ were evaluated by intracytoplasmic staining by flow cytometry. (**c**) Percentage of CD8^+^IFNγ^+^ (n = 11), CD8^+^IL-10^+^ (n = 8), and CD8^+^GzB^+^ (n = 12) cells. Each dot represents a single donor; bars indicate mean ± SEM. A two-tailed Wilcoxon matched-paired test was applied, and statistically significant *p* values are reported. (**d**) Percentage of degranulated GzB^+^CD107a^+^CD8^+^ T cells. Dot plots from one representative donor are presented (left panel)—numbers indicate the percentage of positive cells; dots represent the percentage of GzB^+^CD107a^+^ cells stimulated (+DC) minus the percentage of GzB^+^CD107a^+^ in the unstimulated condition (Alone). Percentage of GzB^+^CD107a^+^CD8^+^ T cells in culture with mDC^IL-10^ (blue dots) and mDC^GFP^ (orange dots) are shown (right panel), each dot represents a single donor (n = 12). A two-tailed Wilcoxon matched-paired test was applied, and statistically significant *p* values are reported. (**e**–**g**) mDC^IL-10^ promoted anergic allo-specific CD8^+^ T cells. (**e**) The percentage of proliferated CD8^+^ T cells in secondary stimulation with the same alloantigen used in priming was evaluated by flow cytometry (n = 14). The proliferation of CD8^+^ T cells upon secondary stimulation with (**f**) mDC in the absence or presence of IL-2 (n = 6), or with (**g**) mDC generated from a third-party donor (n = 12), was evaluated by flow cytometry. The percentage of proliferated CD8^+^ T cells is presented; each dot represents a single donor. a two-tailed Wilcoxon matched paired test was applied; statistically significant *p* values are reported.

We then performed long-term stimulation in which allogeneic CD8^+^ T cells were co-cultured with mDC^IL-10^ or mDC^GFP^ for 14 days (Supplementary Figure S3a). In line with the short-term culture, allogeneic CD8^+^ T cells stimulated with mDC^IL-10^ were significantly (*p* = 0.0005) less activated than CD8^+^ T cells activated with mDC^GFP^, as indicated by the expression of CD25 (28.7 ± 5.7% vs. 66.2 ± 4.2, n = 12), and proliferated at significantly (*p* = 0.0002) lower levels (15.5 ± 2.6% vs. 61.6 ± 6.1%, n = 13) (Figure 2a,b). Moreover, IFNγ and GM-CSF levels in culture supernatants of CD8-mDC^IL-10^ were significantly (*p* = 0.0002 and *p* = 0.0046, respectively) lower compared with those measured in CD8-mDC^GFP^ (0.04 ± 0.008 ng/mL and 0.21 ± 0.065 ng/mL vs. 1.3 ± 0.36 ng/mL, and 0.75 ± 0.15 ng/mL, respectively, n = 13; Supplementary Figure S3b).

Functional characterization of T cells generated with mDC^IL-10^ or mDC^GFP^ was then performed by restimulating cells with the same alloantigen used in priming (Supplementary Figure S3a). As expected, stimulation of allogeneic CD8^+^ T cells with mDC^GFP^ resulted in the induction of allo-specific IFNγ-producing cells (5.7 ± 2.30% vs. 0.94 ± 0.16%, in T(mDC^GFP^) stimulated with mDC vs. T(mDC^GFP^) cultured alone, n = 13, *p* = 0.0007; Figure 2c). Interestingly, although at a lower extent compared with T(mDC^GFP^) cells, restimulation of T(mDC^IL-10^) cells with mDC resulted in a significantly higher (*p* = 0.0344) frequency of IFNγ^+^ cells compared with unstimulated T(mDC^IL-10^) cells (2.2 ± 0.64% vs. 1.4 ± 0.47%, in T(mDC^IL-10^) stimulated with mDC vs. T(mDC^IL-10^) cultured alone, n = 13; Figure 2c), indicating that allogeneic CD8^+^ T cell priming by mDC^IL-10^ occurred, but that upon restimulation, T(mDC^IL-10^) cells contained a lower frequency of allo-specific IFNγ^+^ cells compared with T(mDC^GFP^) cells (Figure 2c). No major differences were observed at steady state and upon mDC stimulation in the percentages of allo-specific T(mDC^IL-10^) and T(mDC^GFP^) cells producing IL-10 (Figure 2c).

We then assessed the cytotoxic activity (GzB expression) of T(mDC^IL-10^) cells in comparison with T(mDC^GFP^) cells (Supplementary Figure S3a). Upon restimulation, the frequency of GzB^+^ cells in T(mDC^IL-10^) cells did not increase compared with that in T(mDC^IL-10^) cultured alone (23.7 ± 3.8% vs. 24.9 ± 3.6%, n = 11), whereas it increased significantly (*p* = 0.0010) in T(mDC^GFP^) (42.1 ± 7.6%, vs. 26.8 ± 5.2% in T(mDC^GFP^) stimulated with mDC vs. T(mDC^GFP^) cultured alone, n = 11; Figure 2c). Moreover, the percentage of T(mDC^IL-10^) cells actively degranulating (GzB^+^CD107a^+^ cells) was significantly (*p* = 0.0005) lower compared with that observed in T(mDC^GFP^) cells (1.2 ± 0.5% vs. 10 ± 2.14%, n = 12; Figure 2d), indicating that stimulation of allogeneic CD8^+^ T cells with IL-10-engineered DC prevents the induction of allo-specific cytotoxic T cells. 

The proliferative capacity of DC^IL-10^-primed T cells in secondary MLR showed that T(mDC^IL-10^) cells proliferated at significantly (*p* = 0.0006) lower levels compared with T(mDC^GFP^) cells (12.3 ± 3.3% vs. 30.3 ± 6.3%, respectively, n = 14; Figure 2e). The lower proliferative capacity was neither associated with the induction of T regulatory cells nor with the up-regulation of activation- and exhaustion-associated markers on DC^IL-10^-stimulated CD8^+^ T cells. Indeed, similar percentages of cells expressing LAG-3 (19.9 ± 4% vs. 24.1 ± 4.96%, n = 14), TIGIT (4.3 ± 1.99% vs. 2.2 ± 0.9%, n = 14), KLRG1 (38.5 ± 8.2% vs. 24 ± 8.1%, n = 9), and TIM-3 (10 ± 2.5% vs. 23.0 ± 5.9%, n = 10) (Supplementary Figure S3c) were observed in T(mDC^IL-10^) and T(mDC^GFP^) cells, respectively. Interestingly, allo-specific T cell hypo-proliferation observed in T(mDC^IL-10^) cells was reverted by the addition of exogenous IL-2 (12 ± 5.6% vs. 40.6 ± 13.6%, n = 6, *p* = 0.0312, Figure 2f), suggesting the induction of anergic cells. The low proliferative capacity upon secondary stimulation was allo-specific, since T(mDC^IL-10^) cells proliferated at similar levels compared with T(mDC^GFP^) cells when stimulated with mDC differentiated from a third-party donor (37.2 ± 5% vs. 37.2 ± 6.8%, n = 12; Figure 2g). 

Overall, these findings indicate that mDC^IL-10^ induce allo-specific anergic CD8^+^ T cells in vitro.

## 3. Discussion

In the present study, we showed that DC^IL-10^ limit the activation and proliferation of allogeneic CD8^+^ T cells. Moreover, allogeneic CD8^+^ T cells primed with DC^IL-10^ are anergic, secrete low levels of the pro-inflammatory cytokines IFNγ and GM-CSF, and have limited cytotoxic activity upon secondary stimulation, while maintaining responsiveness to unrelated allo-antigens. The anergic phenotype of DC^IL-10^-primed CD8^+^ T cells is not associated with the expression of inhibitory molecules. Overall, these findings indicate that DC^IL-10^ are effective in modulating allo-specific CD8^+^ T cell responses in vitro.

In addition to TCR engagement (signal I), optimal CD8^+^ T cell activation requires costimulatory signaling (signal 2) [29] and pro-inflammatory cytokines (signal 3) [27]. DC^IL-10^, at steady state and upon LPS activation, efficiently supplied signal 1 to CD8^+^ T cells since they expressed HLA class I molecules at high and comparable levels to control DC. Moreover, DC^IL-10^ expressed CD86, albeit at variable levels, while its expression on LPS activated (m)DC^IL-10^ was sustained and comparable with control DC. Despite the difference in CD86 expression, both DC^IL-10^ and mDC^IL-10^ elicited activation and proliferation of allogeneic CD8^+^ T cells at comparable levels, indicating that in our culture condition, DC^IL-10^ provide an efficient signal 2, although to a lower extent compared with control DC. The overall inhibition of allogeneic CD8^+^ T cell responses induced by DC^IL-10^ is therefore mediated by the cytokine context during priming, which, in our culture condition, is enriched in IL-10.

The effects of IL-10 on CD8^+^ T cell responses differ according to the cytokine concentration: IL-10 at low concentration modulates CD8^+^ T cells in terms of proliferation and cytotoxic activity, while at high concentration, it stimulates CD8^+^ T cell responses and promotes CD8^+^ T cell expansion in vitro and in vivo [30,31,32,33,34,35]. It has been previously demonstrated that IL-10 has no direct inhibitory effects on the proliferation of CD8^+^ T cells activated by anti-CD3 mAb, but it inhibits alloantigen-specific proliferative responses and induces long-lasting anergy in CD8^+^ T cells indirectly in the presence of antigen-presenting cells in vitro [32]. In line with these findings, IL-10 produced by DC^IL-10^ was effective in reducing activation, proliferation, and cytotoxic activity in allogeneic CD8^+^ T cells. We recently reported that the secretion of IL-10 by engineered DC during CD8^+^ T cell priming is required for modulating T-cell activation and proliferation, while addition of exogenous IL-10 during DC-mediated priming is not effective (Passeri L. et al. [36], under revision). Thus, our findings indicate that inhibition of allogeneic CD8^+^ T cell responses by DC^IL-10^ required allo-Ag presentation in the presence of IL-10. In addition to secreting high levels of IL-10, DC^IL-10^ express the immunomodulatory molecules HLA-G and ILT4, which define their tolerogenic potency [13]. CD8^+^ T cells express ILT2, a known ligand of HLA-G [37]; thus, it can be speculated that the HLA-G/ILT2 interaction might be also implicated in DC^IL-10^-mediated inhibition of CD8^+^ T cell responses. 

The role of the PD1/PDL-1 interaction in modulating T-cell activation and function has been widely characterized, especially in the context of cancer cells [38]. Recently, Li and colleagues [39] demonstrated that the activation of PD-1 signaling during the early phase of Ag recognition disrupts the interaction between the TCR, the MHC/peptide complex and CD8, resulting in the suppression of CD8^+^ T cell function. DC^IL-10^ expresses high levels of PDL-1 (Passeri L. et al. [36], under revision); thus, we cannot exclude that the suppression of allogeneic CD8^+^ T cells observed in our culture conditions might be the results of synergic effects of high levels of IL-10 and a PDL-1/PD-1 interaction during Ag-recognition by CD8^+^ T cells. 

Exogenous or tolerogenic DC-derived IL-10 limit CD4^+^ T cell responses by inducing T cell hypo-responsiveness and anergy [5,11,40,41]. Moreover, stimulation of allo-specific CD8^+^ T cells with IL-10-treated DC resulted in hypo-responsiveness and induction of anergic allo-specific cells [41]. We showed that long-term stimulation of allogeneic CD8^+^ T cells with mDC^IL-10^ induced allo-specific T cell anergy, which can be reverted by addition of exogenous IL-2, thus confirming the ability of IL-10-secreting DC to promote CD8^+^ T cell anergy in vitro. In line with previous studies [41], mDC^IL-10^-primed anergic CD8^+^ T cells failed to degranulate upon encountering allo-specific target cells. Gradual functional impairment of cytotoxic CD8^+^ T cells have been associated with T-cell exhaustion, which is characterized by a loss of pro-inflammatory cytokine production, decreased proliferative capacity, cytotoxic potential [42], and sustained high expression of multiple inhibitory receptors such as PD-1, TIM-3, LAG-3, and TIGIT [43]. Although DC^IL-10^-modulated CD8^+^ T cells secreted lower levels of IFNγ and GM-CSF and proliferated significantly less compared with mDC-primed CD8^+^ T cells, they did not show up-regulated levels of T-cell exhaustion markers. Overall, our findings support the role of DC^IL-10^ in actively inducing tolerogenic phenotype and functions in CD8^+^ T cells.

## 4. Materials and Methods

### 4.1. Vector Production and Titration

VSV-G-pseudotyped third generation bidirectional Lentiviral Vectors (bdLV) encoding human IL-10 and ΔNGFR or green fluorescent protein (GFP) and ΔNGFR were produced by calcium phosphate transfection into 293T cells and concentrated by ultracentrifugation as described previously [44]. Titer was estimated by limiting dilution: vector particles were measured by HIV-1 Gag p24 Ag immune capture (NEN Life Science Products, MA, USA), and vector infectivity was calculated as the ratio between titer and total particles. Titers ranged between 5 × 10^8^ and 6 × 10^9^ transducing units/mL, while infectivity ranged between 5 × 10^4^ and 10^5^ transducing units/ng. To produce concentrated Vpx-incorporating viral-like particles (VLPs), 293T cells were co-transfected with a VSV-G expressing plasmid and the Simian Immunodeficiency Virus-derived packaging plasmid SIV3+, as previously described [45].

### 4.2. Dendritic Cell Differentiation

Human peripheral blood was obtained from healthy donors in accordance with local committee approval (TIGET09) and the Declaration of Helsinki. Peripheral blood mononuclear cells (PBMC) were isolated by density gradient centrifugation over Lympholyte^®^-H (Cederlane, Burlington, Canada). CD14^+^ cells were isolated from PBMC by positive selection using CD14 MicroBeads (Miltenyi Biotech, Bergish Gladbach, Germany) according to the manufacturer’s instructions. Monocytes were exposed for 3 h to Vpx-VLP and then were transduced with bdLV-IL-10 (DC^IL-10^) or with bdLV-GFP (DC^GFP^) at a Multiplicity of Infection (MOI) of 10 as previously described [13]. Cells were cultured in RPMI 1640 (Lonza, Verviers, Belgium) with 10% fetal bovine serum (FBS) (Euroclone, Pero, Italy), 100 U/mL penicillin/streptomycin (Euroclone, Pero, Italy) and 2 mM L-glutamine (Euroclone, Pero, Italy) at 10^6^ cells/mL in a 1 mL volume in a 24-well culture plate. The media was supplemented with rhGM-CSF (Miltenyi Biotech, Bergish Gladbach, Germany) at 100 ng/mL and with rhIL-4 (Miltenyi Biotech, Bergish Gladbach, Germany) at 10 ng/mL and cultured for 7 days at 37 °C with 5% CO_2_. Matured DC^IL-10^ (mDC^IL-10^) and mDC^GFP^ were obtained by activation at day 6 with 1 μg/mL of LPS (Sigma-Aldrich, St. Louis, MO, USA). As expected, we reached an average of transduction, evaluated by ΔNGFR expression, above 80% for all the DC^IL-10^ (matured or not) and mDC^GFP^. From some donors, un-transduced LPS activated DC (mDC) were differentiated. All DC, transduced or not, were harvested on day 7 for phenotypical and functional analyses.

### 4.3. CD8^+^ T Cell Isolation and Culture

CD8^+^ T cells were purified from PBMC by negative selection using a human CD8 T cell isolation kit (Miltenyi Biotech, Bergish Gladbach, Germany) according to the manufacturer’s instructions. T cell cultures were performed in X-VIVO 15 (Lonza, Verviers, Belgium) supplemented with 5% human serum (Sigma Aldrich, Burlington, Massachusetts, USA) and 100 U/mL penicillin/streptomycin (Lonza, Verviers, Belgium). T cells were cultured with 10^4^ allogeneic DC^IL-10^, mDC^IL-10^, or mDC^GFP^ at a 10:1 ratio. After 5 days of stimulation (short-term culture), T cells were collected, washed, and phenotypically and functionally analyzed. For long-term culture experiments, T cells were cultured with allogeneic mDC^IL-10^ (T(mDC^IL-10^)) or mDC^GFP^ (T(mDC^GFP^)) at a 10:1 ratio. At day 3, 1 ng/mL of recombinant human IL-15 (R&D System, Minneapolis, MN, USA) was added. At day 14, cells were collected, washed, and analyzed. In some experiments, CD8^+^ T cells were labelled with Cell Proliferation Dye eFluor^®^ 670 (eBioscience, San Diego, CA, USA) according to the manufacturer’s instructions and analyzed by flow cytometry for their proliferation at the end of the T:DC co-culture.

### 4.4. CD8^+^ T Cell Intracytoplasmic Staining

CD8^+^ T cells were plated for 6 h alone or in the presence of LPS-matured un-transduced DC (mDC) from the same donor used for priming at a 10:1 ratio. A CD107a fluorophore-conjugated antibody was added immediately after cell seeding, and 10 μg/mL of Brefeldin A (Sigma-Aldrich, St. Louis, MO, USA) was added for the last 3 h of culture. Subsequently, cells were stained with LIVE/DEAD^TM^ Fixable Dead Cell Stain Kit (Invitrogen, Carlsbad, CA, USA), anti-CD3 (Becton Dickinson, Franklin Lakes, NJ, USA), and anti-CD8 (Becton Dickinson, Franklin Lakes, NJ, USA), and fixed with Dulbecco’s Phosphate-Buffered Saline (DPBS, Corning, Manassas, VA, USA) containing 2% formaldehyde solution (Thermo Fisher Scientific, Waltham, MA, USA). Intracellular cytokine staining was performed permeabilizing cells with Saponin 0.5% (Sigma-Aldrich, St. Louis, MO, USA), and staining was performed with anti-IFNγ, anti-IL-10, anti-Granzyme (Gz)B, and anti-Perforin (Prf) fluorochrome-conjugated antibodies (all from BD Bioscience, Franklin Lakes, NJ, USA).

### 4.5. CD8^+^ Secondary Stimulation (II MLR)

For the recall response, T cells primed with mDC^IL-10^ (T(mDC^IL-10^)) or with mDC^GFP^ (T(mDC^GFP^)) were stained with Cell Proliferation Dye eFluor^®^ 450 (eBioscience, San Diego, CA, USA) and plated for an additional 4 days in the presence of mDC from the same donor used for priming at a T:DC ratio of 10:1 with or without 100 U/mL IL-2 (Proleukin, Novartis, Basel, Switzerland). For some donors, T(mDC^IL-10^) and T(mDC^GFP^) cells were restimulated with mDC differentiated from a third-party donor for 4 days. At the end of the culture, the proliferation of CD8^+^ T cells was analyzed by flow cytometry.

### 4.6. Cytokine Determination

For DC, at the end of the 7 days of differentiation, cells were collected, washed, and plated in RPMI complete medium at a concentration of 1 M/mL. Supernatants were collected after 48 h and levels of IL-10 were evaluated. For CD8^+^ T cells, IFNγ and GM-CSF production was quantified in co-culture supernatants. Cytokine quantification was performed by standard sandwich ELISA with purified and biotinylated antibody couples (Becton Dickinson, CA, USA). The limits of detection were 31 pg/mL for IFNγ and GM-CSF, and 15 pg/mL for IL-10.

### 4.7. Flow Cytometry

Fluorochrome-conjugated antibodies against the following antigens were used for human DC staining: NGFR, CD1a, CD14, CD86, CD16, CD163 (Becton Dickinson, Franklin Lakes, NJ, CA, USA), CD141 (Miltenyi Biotech, Bergish Gladbach, Germany), HLA-G (Exbio, Praha, Czech Republic), ILT4 (Beckman Coulter, Jersey City, NJ, USA), and HLA-ABC+ (Biolegend, San Diego, CA, USA). The following fluorochrome-conjugated antibodies were used for CD8^+^ T cell staining: anti-CD3, anti-CD4, anti-CD8, anti-CD45RA, anti-CD25, anti-CD71, anti-CD137 (Becton Dickinson, Franklin Lakes, NJ, USA), anti-TIGIT (Invitrogen, Carlsbad, CA, USA), anti-KLRG1, anti-TIM-3 (Biolegend, San Diego, CA, USA), and anti-LAG3 (Miltenyi Biotech, Bergish Gladbach, Germany). FcR Blocking Reagent (Miltenyi Biotech, Bergish Gladbach, Germany) was used in all preparations to avoid non-specific staining. Briefly, cells were centrifuged and resuspended in DPBS (Corning, Manassas, VA, USA) supplemented with 2% FBS (Lonza, Verviers, Belgium). Cells were stained with LIVE/DEAD^TM^ Fixable Dead Cell Stain Kit (Invitrogen, Carlsbad, CA, USA) and antibody mix and incubated at room temperature for 15 min, centrifuged, and fixed with DPBS (Corning, Manassas, VA, USA) containing 1% formaldehyde solution (Thermo Fisher Scientific, Waltham, MA, USA).

Samples were acquired using a CytoFlex LX flow cytometer (Becton Dickinson, Mountain View, CA, USA), and data were analyzed with FCS express 7 (De Novo Software, Glendale, CA, USA). Quadrant markers were set according to the relative fluorescence minus one (FMO) staining.

### 4.8. Statistical Analysis

Friedman matched-pairs test, in association with Dunn’s multiple comparison test, was applied in Figure 1. Wilcoxon matched-pairs test (two-tailed) was used for statistical analysis in Figure 2 and in Supplementary Figures S1–S3. Statistically significant *p* values are reported in all the figures. All results are presented as mean values ± SEM, unless differently specified in the figure legend. Results were analyzed using GraphPad Prism 9.0 (GraphPad Software, San Diego, CA, USA).

## 5. Conclusions

In the context of allo-transplantation, recipient T cells can recognize donor allo-Ags and give rise to an inflammatory immune response leading to graft rejection [46]; several studies have highlighted the major role played by cytotoxic CD8^+^ T cells in allogeneic responses (reviewed in [47]). In the present study, we reported that DC^IL-10^ effectively inhibit CD8^+^ T cell effector function and actively promote allogeneic anergic CD8^+^ T cells in vitro. These findings, in addition to their previously reported ability to modulate allo-specific CD4^+^ T cell responses and to promote Tr1 cells [13], support the conclusion that IL-10-engineered DC represent a promising tool for developing cell-based therapies to promote/restore tolerance in transplantation settings. Further studies in pre-clinical models of allo-transplantation are warranted to better define the feasibility and efficacy of DC^IL-10^ cell therapy.

## Data Availability

The data that support the findings of this study are available from the corresponding author upon reasonable request.

## Supplementary Materials

The following supporting information can be downloaded at: https://www.mdpi.com/article/10.3390/ijms24119128/s1.
