# Supplementary material for: IL-10-Engineered Dendritic Cells Modulate Allogeneic CD8+ T Cell Responses
Source: Int J Mol Sci. 2023 May 23;24(11):9128. doi: 10.3390/ijms24119128 (PMC10252493; doi:10.3390/ijms24119128)
Supplement: Supplementary file 1 [file ijms-24-09128-s001.zip › ijms-2377711-supplementary.pdf]

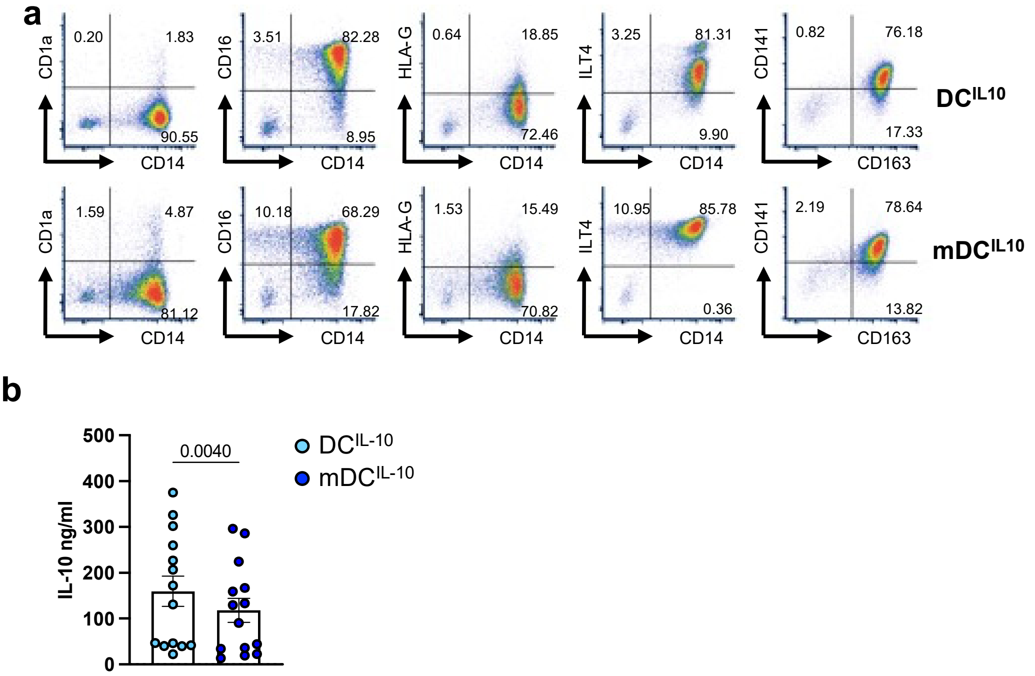

Supplementary Figure S1. DC^{IL-10} and mDC^{IL-10} have a comparable tolerogenic phenotype. CD14^{+} cells isolated from peripheral blood of healthy subjects were transduced during DC differentiation with LV-IL-10 and left unstimulated (DC^{IL-10}) or activated with LPS (mDC^{IL-10}). At the end of the differentiation, (**a**) the expression of the indicated markers was evaluated by flow cytometry; and (**b**) the release of IL-10 in culture supernatant was assessed by ELISA. Each dot represents a single donor (n=14), bars indicate mean±SEM. Two-tailed Wilcoxon matched paired test was applied, statistically significant P values are reported.

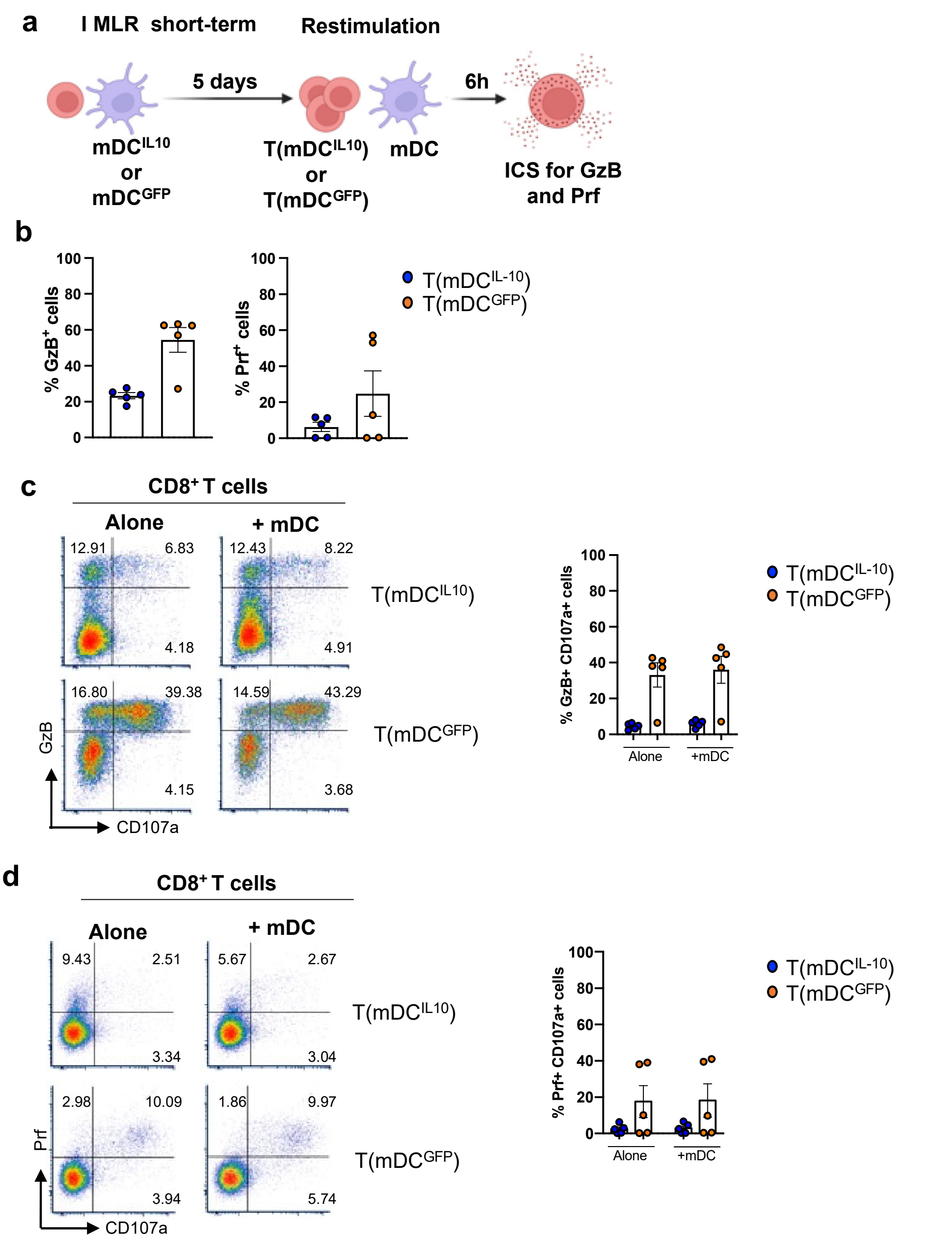

Supplementary Figure S2. Degranulation activity of alloreactive CD8^{+} T cells stimulated with mDC^{IL-10} and mDC^{GFP} in short term co-culture. CD14^{+} cells isolated from peripheral blood of healthy subjects were transduced with LV-IL-10 or LV-GFP and activated with LPS (mDC^{IL-10} and mDC^{GFP}, respectively). (**a**) Experimental design: differentiated DC were used to stimulate at 10:1 (T:DC) ratio allogeneic CD8^{+} T cells isolated from peripheral blood of healthy subjects for 5 days (I MLR, short-term). After culture, CD8^{+} T cells generated with mDC^{IL-10} T(mDC^{IL-10}) or mDC^{GFP} T(mDC^{GFP}) were left unstimulated or restimulated for 6 hours with mDC generated from the same donor used for priming. Degranulation was assessed by intracytoplasmic staining (ICS) for granzyme B (GzB) and perforin (Prf). (**b-d**) mDC^{IL10} modulated the expression of GzB and Prf in allogeneic CD8^{+} T cells. (**b**) Percentages of CD8^{+}GzB^{+} and CD8^{+}Prf^{+} cells were assessed by flow cytometry. Each dot represents a single donor (n=5), bars indicate mean±SEM. (**c**) Percentages of GzB^{+}CD107a^{+} cells evaluated by flow cytometry after 6 hours of activation with mDC are shown. Dot plots from one representative donor are presented (left panel), numbers indicate percentage of positive cells. Each dot represents a single donor (n=5), bars indicate mean±SEM. (**d**) Percentages of Prf^{+}CD107a^{+} cells evaluated by flow cytometry after 6 hours of activation with mDC. Each dot represents a single donor (n=5), bars indicate mean±SEM.

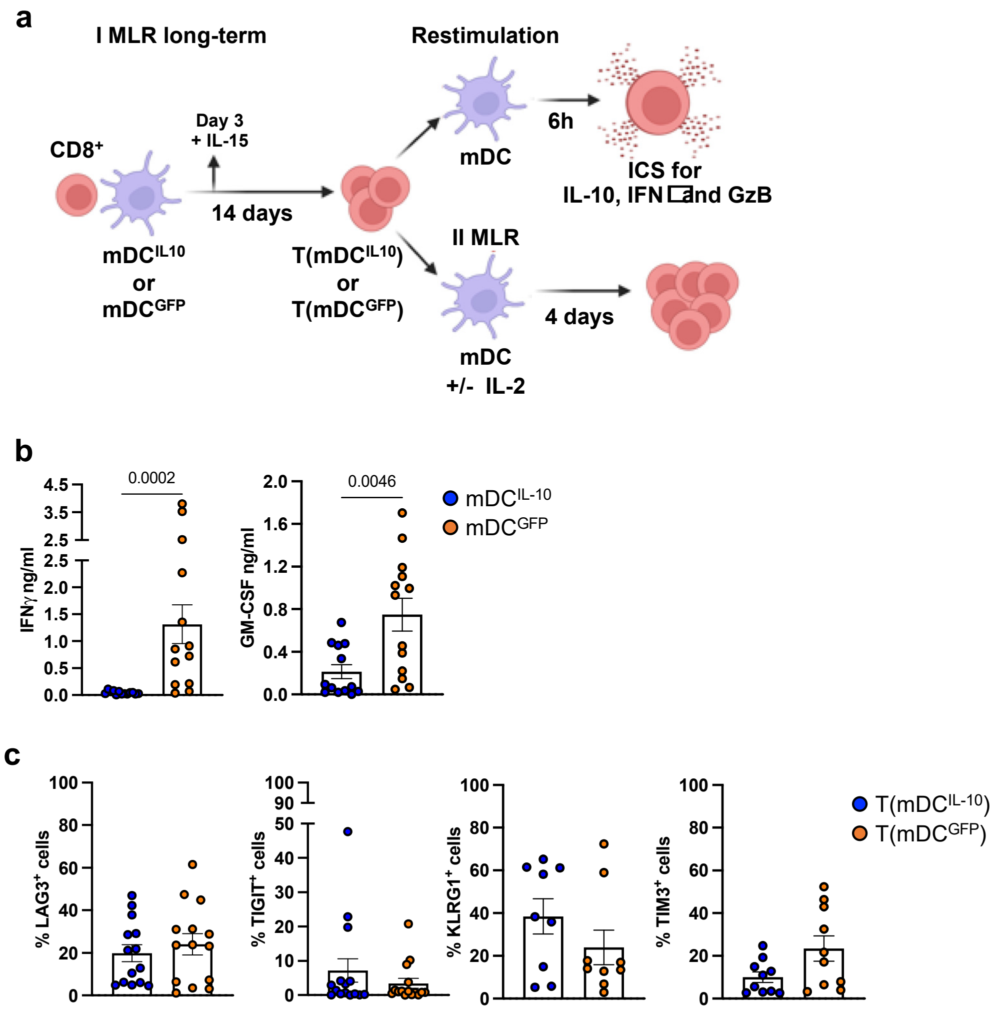

Supplementary Figure S3. mDC^{IL-10} do not induce exhausted CD8$^+$ T cells. CD14$^+$ cells isolated from peripheral blood of healthy subjects were transduced with LV-IL-10 or LV-GFP and activated with LPS (mDC^{IL-10} and mDC^{GFP}, respectively). (**a**) Experimental design: differentiated DC were used to stimulate at 10:1 (T:DC) ratio allogeneic CD8$^+$ T cells for 14 days (I MLR long-term). After culture, CD8$^+$ T cells were left unstimulated or restimulated for 6 hours with mature DC (mDC) generated from the same donor used for priming, and levels of IL-10, IFNγ and granzyme B (GzB) were assessed by intracytoplasmic staining (ICS). In parallel, CD8$^+$ T cells were stained with proliferation dye and co-cultured for 4 days (II MLR) with mDC (at 10:1 ratio) generated from the same donor used for priming in presence or absence of IL-2. (**b**) after 14 days of culture, IFNγ and GM-CSF were evaluated by ELISA in cell culture supernatants, each dot represents a single donor (n=13), bars indicate mean±SEM. Two-tailed Wilcoxon matched paired test was applied, statistically significant P values are reported. (**c**) mDC^{IL-10}-stimulated CD8$^+$ T cells do not express markers of exhaustion. The percentage of CD8$^+$ T cells expressing the indicated exhaustion markers was evaluated by flow cytometry. Each dot represents a single donor, bars indicate mean±SEM. Two-tailed Wilcoxon matched paired test was applied, statistically significant P values are reported.
